# *Pulicaria glutinosa* Extract: A Toolbox to Synthesize Highly Reduced Graphene Oxide-Silver Nanocomposites

**DOI:** 10.3390/ijms16011131

**Published:** 2015-01-05

**Authors:** Abdulhadi H. Al-Marri, Mujeeb Khan, Merajuddin Khan, Syed F. Adil, Abdulrahman Al-Warthan, Hamad Z. Alkhathlan, Wolfgang Tremel, Joselito P. Labis, Mohammed Rafiq H. Siddiqui, Muhammad N. Tahir

**Affiliations:** 1 Department of Chemistry, College of Science, King Saud University, P.O. Box 2455, Riyadh 11451, Saudi Arabia; E-Mails: okor-999@hotmail.com (A.H.A.-M.); kmujeeb@ksu.edu.sa (Mu.K.); mdk.chem@gmail.com (Me.K.); sfadil@ksu.edu.sa (S.F.A.); awarthan@ksu.edu.sa (A.A.-W.); khathlan@ksu.edu.sa (H.Z.A.); 2 Institute of Inorganic and Analytical Chemistry, Johannes Gutenberg-University of Mainz, Mainz 55122, Germany; E-Mail: tremel@uni-mainz.de; 3 King Abdullah Institute for Nanotechnology, King Saud University, Riyadh 11451, Saudi Arabia; E-Mail: jlabis@ksu.edu.sa

**Keywords:** graphene, silver nanoparticles, plant extract, nanocomposites, surface enhance Raman scattering (SERS)

## Abstract

A green, one-step approach for the preparation of graphene/Ag nanocomposites (PE-HRG-Ag) via simultaneous reduction of both graphene oxide (GRO) and silver ions using *Pulicaria glutinosa* plant extract (PE) as reducing agent is reported. The plant extract functionalizes the surfaces of highly reduced graphene oxide (HRG) which helps in conjugating the Ag NPs to HRG. Increasing amounts of Ag precursor enhanced the density of Ag nanoparticles (NPs) on HRG. The preparation of PE-HRG-Ag nanocomposite is monitored by using ultraviolet–visible (UV-Vis) spectroscopy, powder X-ray diffraction (XRD), and energy dispersive X-ray (EDX). The as-prepared PE-HRG-Ag nanocomposities display excellent surface-enhanced Raman scattering (SERS) activity, and significantly increased the intensities of the Raman signal of graphene.

## 1. Introduction

Graphene, a single layer honey-combed network of sp^2^ hybridized carbon atoms, has attracted tremendous attention of the scientific community due to its unique two-dimensional structure and its extraordinary physicochemical, optical and electronic properties [1,2,3]. Graphene has been exploited in various fields, including sensing, energy storage and catalysis [4,5,6]. Graphene based nanocomposites of metallic nanoparticles (NPs) combining the properties of both the components in synergistic manner have been used for various purposes, e.g., for chemical and biological sensors, as energy storage materials and effective catalysts [7,8,9,10]. Moreover, nanocomposites containing graphene- with metal and surface bound metal oxide NPs have been extensively applied for various applications, such as, thermal interface materials, catalysts, adsorbent materials [11,12,13,14,15,16], surface-enhanced Raman scattering (SERS) substrates and so on [17,18]. SERS enhances the signal intensity by orders of magnitudes, and has been potentially exploited for the ultra-sensitive detection of various analytes, including a number of chemical and biological molecules [19,20]. Among the precious metal NPs, which are available with good control on size and morphology, silver (Ag) NPs have high SERS activity and have been widely applied [21,22]. Significant efforts have been made to prepare graphene silver (Ag) nanocomposites (HRG-Ag), combining the properties of Ag and graphene, e.g., high SERS activity of silver and large specific surface area of graphene [23,24].

In order to synthesize uniform and stable dispersions of graphene-inorganic nanoparticles based hybrid materials requires the surface stabilization of as synthesized GO using surface functionalization [25]. There are generally two different approaches to synthesize graphene/NPs composites: (i) pre-synthesized NPs added on the surface of the prefunctionalized graphene oxide (GRO) sheets followed by chemical reduction to obtain nanocomposites [26]; (ii) The NPs can be directly grown on the surface of the HRG nanosheets reduced separately using inorganic precursors [27,28]. HRG-Ag nanocomposites obtained via route (i) usually suffer from poor stability and reproducibility, due to the aggregation of graphene layers, which seriously effects the properties of the nanocomposites [29].

Significant efforts have been made to prepare HRG-Ag nanocomposites in a single step via *in situ* reduction of both GRO and Ag salts using various reducing agents [30,31]. However, some external stabilizers were used to maintain the stability of the dispersion and to control the size and shape of Ag NPs [32]. In most of these cases hazardous or toxic reducing agents and chemical stabilizers, such as NaBH_4_, formaldehyde, hydrazine, poly-(*N*-vinyl-2-pyrrolidone) were used for the reduction and stabilization of the composite materials, which imposes serious environmental risks and limits the applications of the hybrid materials [33]. Therefore, environmental friendly, economically viable, stable and energy efficient synthesis of HRG-Ag nanocomposites is highly desirable [34,35].

To establish environmentally friendly synthetic protocols (green synthesis) for metallic NPs using various biological materials, plant extracts have attracted attention as reducing agents due to the fact that they are cheap and relatively easy to handle [36,37]. In a previous study, we have demonstrated the synthesis of Ag NPs using *Pulicaria glutinosa* plant extract both as reducing as well as stabilizing agent [38,39]. Furthermore, we have also demonstrated the synthesis of highly reduced graphene oxide (PE-HRG) by a facile and efficient reduction of graphene oxide using the same *P. glutinosa* plant extract [40]. Here we extend our work to develop a facile, single step, green chemistry protocol for the synthesis of HRG-Ag nanocomposites from GRO and AgNO_3_ using *P. glutinosa* plant extract acting both as reducing agent and *in situ* functionalization ligand to bind Ag NPs onto HRG sheets. The as-prepared HRG-Ag nanocomposites were characterized by X-ray powder diffraction (XRD), Fourier-transform infrared spectroscopy (FT-IR), ultraviolet–visible absorption (UV-Vis) spectroscopy, and transmission electron microscopy (TEM). Furthermore, the SERS activity of the as-prepared HRG-Ag nanocomposites was analyzed.

## 2. Results and Discussion

**Scheme 1:**
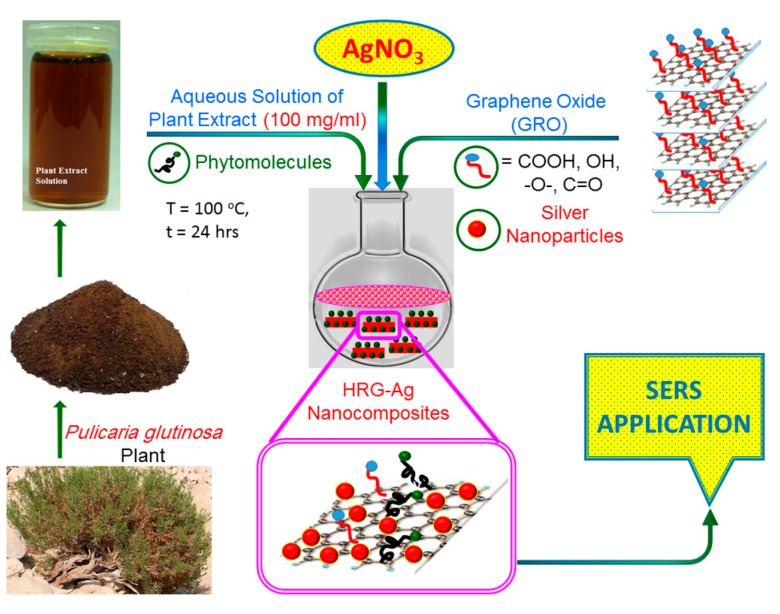
Schematic illustration of the green synthesis of graphene/silver nanocomposites (PE-HRG-Ag) using aqueous extract of *P. glutinosa*.

The green, one pot synthesis with *P. glutinosa* plant extract as reducing and stabilizing agent is a facile and environmentally friendly approach to synthesize HRG-Ag nanocomposites. The preparation of plant extract and synthesis of HRG-Ag nanocomposites is shown in Scheme 1. Briefly, plant extract (PE) was added to the dispersion of graphene oxide (GRO) and AgNO_3_ and allowed to stir under reflux for 24 h. The color of the dispersion gradually changed from dark brown to black after addition of the plant extract (PE), indicating the formation of PE-HRG-Ag. It is worth mentioning that no color change was observed even after 72 h when reaction was performed under similar set of conditions but without adding PE. Apparently, the preparation of HRG-Ag nanocomposites is facilitated by the anti-oxidant activity of *P. glutinosa* PE, which was confirmed in our previous study [39]. *P. glutinosa* PE is rich in flavonoids, polyphenols and other phytomolecules, which were mainly responsible for the simultaneous reduction of Ag ions and graphene oxide during the preparation of HRG-Ag nanocomposites [40]. Furthermore, to study the effect of the concentration of AgNO_3_ on the size, morphology, covering density of Ag NPs on HRG and also on their Raman applications, two different samples of PE-HRG-Ag were prepared with increasing concentration of AgNO_3_ e.g., PE-HRG-Ag-1 and 2 were prepared with AgNO_3_ concentrations 0.5 mmol AgNO_3_ and 1 mmol AgNO_3_ while keeping the amounts of GRO and PE constant.

**Figure 1:**
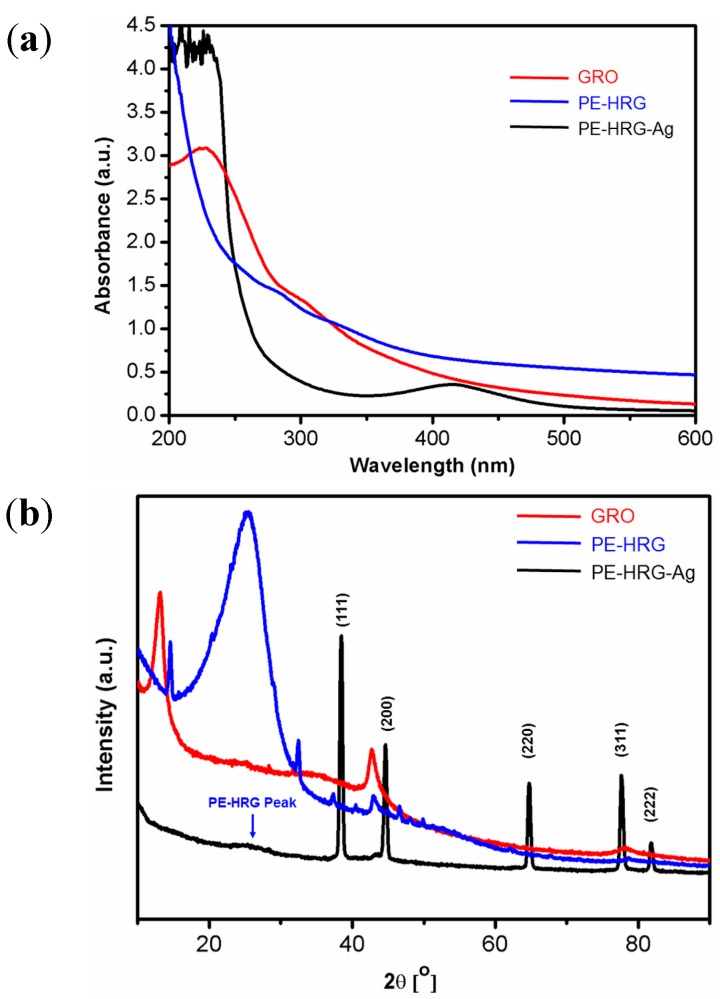
(**a**) Ultravoilet–visible (UV-Vis) absorption spectra of graphene oxide (GRO), plant extract (PE) mediated highly reduced graphene oxide (PE-HRG), graphene/silver nanocomposites (PE-HRG-Ag) prepared by using *P. glutinosa* plant extract (**b**) XRD spectra of graphene oxide (GRO), PE mediated highly reduced graphene oxide (PE-HRG) and graphene/silver (PE-HRG-Ag) nanocomposites prepared by using *P. glutinosa* plant extract.

The formation of PE-HRG-Ag was initially monitored by UV-Vis spectroscopy as shown in Figure 1a. The as prepared GRO shows two characteristic absorption bands centered at ~230 nm and 301 nm (Figure 1a red line). However, after deposition of the Ag NPs on the surface of graphene by *in situ* reduction of GRO and AgNO_3_ using PE, a new band emerged at about ~420 nm corresponding to the characteristic surface plasmon absorption band of Ag NPs [38]. The disappearance of the characteristics peaks of GRO and the emergence of a new band corresponding to the Ag NPs clearly indicates a simultaneous reduction of both GRO and AgNO_3_ and the formation of PE-HRG-Ag composite. The crystalline nature of PE-HRG-Ag nanocomposites has been confirmed by XRD analysis (Figure 1b). GRO exhibits a reflection at a low angle (2θ = 10.9°) compared to pristine graphite (2θ = 26.4°). (Figure 1b red line). The reflection at 2θ = 10.9° in PE-HRG disappeared and a new reflection emerged at 2θ = 22.4°, indicating a reduction of GRO (Figure 1b blue line). However, in PE-HRG-Ag apart from the characteristic reflections due to reduced graphene oxide (2θ = 22.4°), five distinct reflections appeared in the diffractogram at 37.50° (111), 44.13° (200), 63.91° (220), 76.89° (311), and 81.13° (222) which correspond to the face-centered cubic structure of the Ag NPs. The absence of any additional reflections besides those of graphene and Ag clearly indicates the reduction of GRO and the Ag ions and also suggests that the PE-HRG-Ag lattice is unaffected by other molecules of the plant extract.

**Figure 2:**
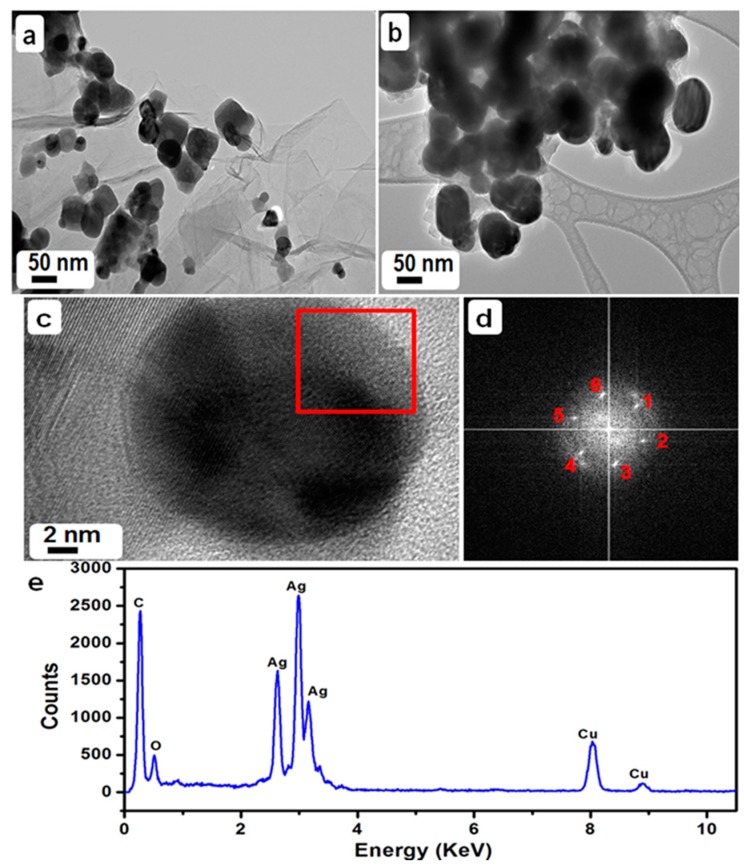
(**a,b**) Overview TEM images of the graphene/silver nanocomposites prepared by using a 50 wt %, 0.5 mmol solution of AgNO_3_ (PE-HRG-Ag-1) and a 100 wt %, 0.5 mmol solution of AgNO_3_ (PE-HRG-Ag-2); (**c**) High resolution transmission electron microscopic (HRTEM) image of a Ag NP; (**d**) FFT measured inside red square in Figure 2c, numbers 1–6 represent important reflections which confirms the cubic structure of Ag NP; and (**e**) Energy dispersive X-ray (EDX) spectrum of the PE-HRG-Ag nanocomposite confirming the presence of Ag and C.

The morphology and structure of PE-HRG-Ag-1 and 2 were analyzed via transmission electron microscopy TEM. Figure 2a,b show TEM images of the as-prepared PE-HRG-Ag-1 and PE-HRG-Ag-2 nanocomposites, with the weight ratio between PE-HRG and Ag NPs of 50 wt % and 100 wt % respectively. In the background, (Figure 2a), TEM images revealed a transparent and sheet like structure for PE-HRG. A large number of wrinkles and scrolls were observed on the surface of the PE-HRG sheet, which remained stable under the high energy electron beam.

The TEM images clearly indicate that the Ag nanoparticles density can be increased by increasing the amount of AgNO_3_. Spherical Ag NPs were firmly attached to the PE-HRG sheets. The average diameter of the Ag NPs was ~50 nm. However, it is worth mentioning that the particle size distribution was more uniform for high concentrations of AgNO_3_. The HRTEM image along with corresponding FFT (taken from red square area) confirmed the spherical morphology as well as face centered cubic (*fcc*) crystal symmetry. The interplanar distances of 0.204 nm correspond to the (002) plane with crystallographic {1 1 1} zone of *fcc* cubic silver. In addition, the elemental composition of the as-prepared PE-HRG-Ag-1 and PE-HRG-Ag-2 was also determined by energy dispersive X-ray analysis (EDX). The intense signal in the EDX spectrum (Figure 2e) clearly indicates the presence of Ag NPs. The other prominent signals in the range from 0.0–0.5 keV represents the presence of carbon and oxygen, which strongly suggests the presence of graphene.

**Figure 3:**
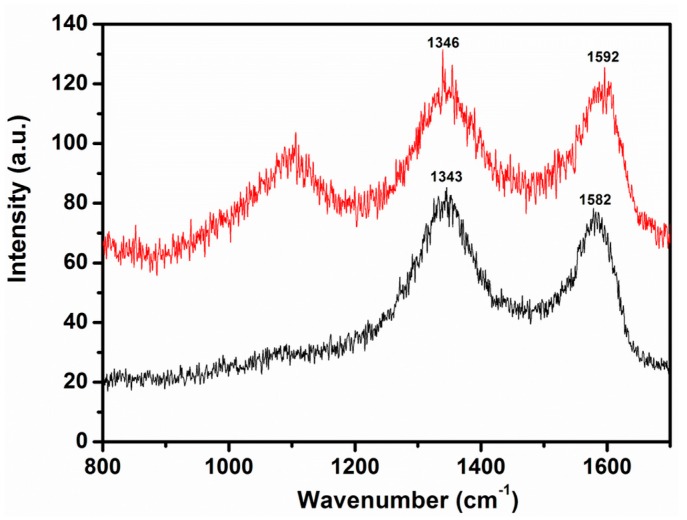
Raman spectra of graphene oxide (GRO, red line) and highly reduced graphene oxide (PE-HRG, black line) using *P. glutinosa* plant extract [41].

Due to their strong SERS effect, Ag NPs significantly enhance the Raman scattering signals of the adsorbed molecules. Therefore, Ag NPs have been successfully applied to enhance the intensities of the Raman signal of CNTs and graphene [33,41]. Typically, graphene exhibits two Raman signals of weak intensities, the G and D bands, which appear at 1575 and 1350 cm^−1^ respectively, which are shifted after oxidation and are located at 1592 and 1346 cm^−1^, due to the destruction of the sp^2^ character and the formation of defects in the sheets caused by the extensive oxidation [40]. After reduction with *P. glutinosa* PE these bands are relocated towards their ideal positions at 1582 and 1343 cm^−1^, which confirms the reduction of GRO, but the intensities of these signals are very weak as shown in Figure 3.

**Figure 4:**
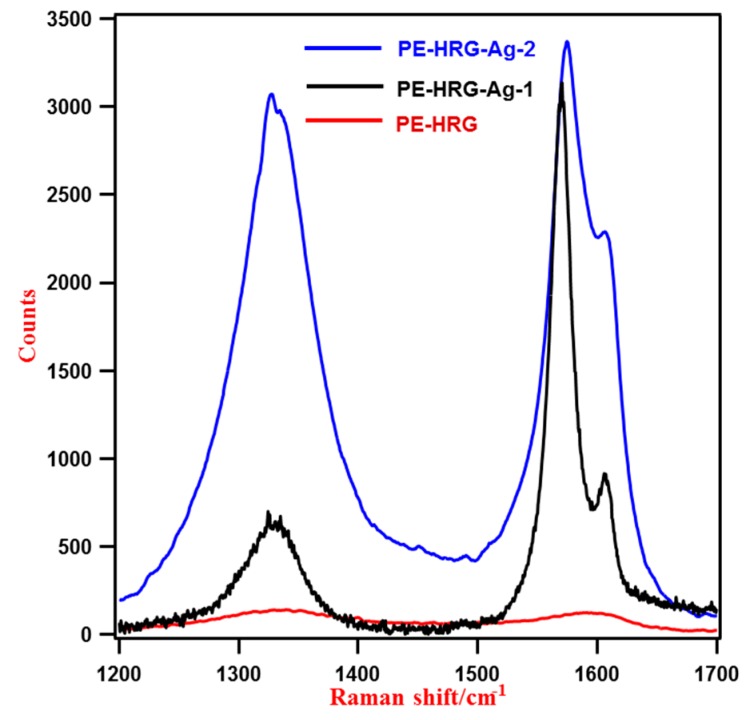
Raman spectra of PE-HRG with and without Ag NPs. With increasing the concentration of Ag NPs the intensities of the Raman signals also increases.

However, after binding Ag NPs on the surface of PE-HRG, the intensities of these signals increased significantly in PE-HRG-Ag compared to the pristine PE-HRG (Figure 4). In the case of PE-HRG-Ag-1 with a lower concentration of Ag NPs (50 wt %) the enhancement factor was calculated to be around 23, and in PE-HRG-Ag-2 (100 wt %) it was estimated to be 31. The enhancement factors can be even further enhanced by increasing the concentration of the Ag NPs. In addition, the Raman spectra exhibit a clear splitting of the peak at 1582 cm^−1^ which is related to the presence of multilayers of graphene and points the deviation from a single layer configuration [42]. The presence of multilayer graphene in PE-HRG-Ag has also been confirmed by the TEM results. Notably, pristine PE-HRG, consist of multilayer graphene, as confirmed by the TEM and AFM analyses [40], however, the splitting of this particular Raman signal is not visible here, due to the lower intensity of the signals.

## 3. Experimental Section

### 3.1. Materials

Graphite powder (99.999%, −200 mesh) was purchased from Alfa Aesar (Haverhill, MA, USA). Concentrated sulfuric acid (H_2_SO_4_ 98%), potassium permanganate (KMnO_4_ 99%), sodium nitrate (NaNO_3_, 99%) and hydrogen peroxide (H_2_O_2_, 30 wt %) and all other organic solvents were obtained from Aldrich chemicals (Steinheim, Germany) and were used without further purification.

The whole plant of wild growing *P. glutinosa* was collected from the hilly area of Al-Hair in central Saudi Arabia during March 2011. The identity of the plant material was confirmed by a plant taxonomist from the Herbarium Division of the College of Science, King Saud University, Riyadh, Kingdom of Saudi Arabia. A voucher specimen was deposited in our laboratory as well as in the Herbarium Division of King Saud University with the voucher specimen number KSU-21598. The details of the preparation of plant extract were given elsewhere [39]. The solution of the plant extract which was used for the reduction of GRO was prepared using 0.1 gram of plant extract in 1 mL of solvent.

### 3.2. Preparation of Graphite Oxide (GO)

Graphite oxide (GO) was synthesized from graphite powder by a modified Hummers method [40,42]. Initially, 2 g of natural graphite and 1.75 g of NaNO_3_ (purity 99%) were taken in a three-neck flask, to which 150 mL of H_2_SO_4_ (98%) was slowly added. The mixture was allowed to stir for 2 h under ice-water, after 2 h, 9 g of KMnO_4_ (99%) were slowly added under constant stirring over a period of 2 h. The remaining mixture was then allowed to react for five days at room temperature. Thereafter, 200 mL of 5 wt % H_2_SO_4_ aqueous solution were added over a period of 1 h, and the solutions was stirred for 2 h. Subsequently, 6 g of 30 wt % H_2_O_2_ aqueous solution were added, and the mixture was left for stirring for another 2 h. The resulting solution was thoroughly washed with an aqueous solution containing 3 wt % H_2_SO_4_ and 0.5 wt % H_2_O_2_ several times and finally three times with deionized water (DI). The resultant mixture was dispersed in DI water and centrifuged for 2 h at 9000 rpm. The resulting dispersion was purified by washing with DI water 20 times to obtain a brown-black homogeneous dispersion.

### 3.3. Preparation of Highly Reduced Graphene Oxide (PE-HRG)

Graphite oxide, GO (200 mg) was dispersed in 40 mL of distilled water and sonicated for 30 min to obtained graphene oxide (GRO) sheets. The resultant suspension was heated to 100 °C. Subsequently 10 mL of an aqueous solution of plant extract (0.1 g/mL) was added, and the suspension was allowed to stir for 24 h at 98 °C. Afterwards, the highly reduced graphene oxide (PE-HRG) was collected by filtration as a black powder. The obtained material was washed with distilled water several times to remove excess plant extract residue and redispersed into water for sonication. The suspension was centrifuged at 4000 rpm for another 30 min, and the final product was collected by vacuum filtration and dried *in vacu*.

### 3.4. Preparation of Highly Reduced Graphene Oxide/Ag Nanocomposites (PE-HRG-Ag)

An aqueous solution of 170 mg of graphene oxide (GRO) and 0.5 mM (84.93 mg) of AgNO_3_ (50 wt % of graphene oxide) were used for the synthesis of PE-HRG-Ag-1 nanocomposites. Initially, 170 mg of GRO was dispersed in 50 mL of water by 30 min of sonication. Subsequently, the reaction mixture was prepared in a 250 mL round bottom flask by dissolving 0.5 mmol of AgNO_3_ in 40 mL of water. To this solution, 50 mL GRO dispersion and 10 mL of an aqueous solution of *P. glutinosa* plant extract were added and the mixture was stirred at 90 °C for 24 h. After 24 h the reaction was stopped and the resultant mixture was washed three times with water using centrifugation. The product was obtained as black powder (185 mg).

### 3.5. Characterization

UV spectra were recorded on a Perkin Elmer lambda 35 (Perkin Elmer, Waltham, MA, USA) UV-Vis spectrophotometer. The analysis was performed in quartz cuvettes using DI water as a reference solvent. The stock solutions of PE-HRG, PE-HRG-Ag and GRO for the UV measurements were prepared by dispersing 5 mg of sample in 10 mL of DI water, which was further sonicated for 30 min. The UV samples for the GRO, PE-HRG and PE-HRG-Ag were prepared by diluting 1 mL of stock solution in 9 mL of water. XRD diffractograms were collected on a Altima IV (Rigaku, Tokyo, Japan) X-ray powder diffractometer using Cu Kα radiation (λ = 1.5418 Å). Transmission electron microscopy (TEM) was performed on a JEOL (Peabody, MA, USA) JEM 1101 microscope. The samples for TEM were prepared by placing a drop of the primary sample on a holy carbon copper grid, and dried for 6 h at 80 °C in an oven. Raman spectral measurements were performed using a Renishaw (Gloucestershire, UK) Raman microscope, equipped with a 514.5 nm line of argon ion laser as excitation source. The laser power at the sample was 8 mW, and the data acquisition time was 20 s.

## 4. Conclusions

In summary, we demonstrate one step, green and environmentally benign method for binding Ag NPs on the surface of the HRG using *P. glutinosa* extract*.* The reduction of the GRO, the Ag ions and the deposition of the Ag NPs was carried out in a single step without using any harmful chemical reagents. The density of Ag NPs on the surface of the graphene can be simply adjusted by varying the AgNO_3_ concentration. During this study, a simple coating of the Ag NPs on graphene has substantially increased the intensity of the graphene Raman signal. Therefore, the as-prepared PE-HRG-Ag nanocomposites have great potential as substrates for SERS activities for the detection of chemical and biological analytes.

## References

[1] Geim A.K., Novoselov K.S. (2007). The rise of graphene. Nat. Mater..

[2] Novoselov K.S., Fal’ko V.I., Colombo L., Gellert P.R., Schwab M.G., Kim K. (2012). A roadmap for graphene. Nature.

[3] Chen Y.B., Liu J.S., Pang L. (2013). Recent trend in graphene for optoelectronics. J. Nanopart. Res..

[4] Mahmood N., Zhang C., Yin H., Hou Y. (2014). Graphene-based nanocomposites for energy storage and conversion in lithium batteries, supercapacitors and fuel cells. J. Mater. Chem. A.

[5] Cooper J.S., Myers M., Chow E., Hubble L.J., Cairney J.M., Peicic B., Müller K.-H., Wieczorek L., Raguse B. (2014). Performance of graphene, carbon nanotube, and gold nanoparticle chemiresistor sensors for the detection of petroleum hydrocarbons in water. J. Nanopart. Res..

[6] Kong X.K., Chen C.L., Chen Q.W. (2014). Doped graphene for metal-free catalysis. Chem. Soc. Rev..

[7] Li L., Liu J., Tan G., Jiang J., Peng S., Deng M., Qian D., Feng Y., Liu Y. (2014). High-sensitivity paracetamol sensor based on Pd/graphene oxide nanocomposite as an enhanced electrochemical sensing platform. Biosens. Bioeletron..

[8] Shen Y., Chen J.S., Zhu J., Yan Q., Hu X. (2013). Growth of two-dimensional ultrathin anatase TiO_2_ nanoplatelets on graphene for high-performance lithium-ion battery. J. Nanopart. Res..

[9] Zhai J., Sun L., Yu H., Li H., Zhang X., Yang H., Xu J. (2014). A facile approach of fabricating graphene-encapsulated ZnO microspheres and their synergic effect on photocatalytic performance. J. Nanopart. Res..

[10] Li Q., Xu P., Zhang B., Tsai H., Wang J., Wang H.L., Wu G. (2013). One-step synthesis of Mn_3_O_4_/reduced graphene oxide nanocomposites for oxygen reduction in nonaqueous Li–O_2_ batteries. Chem. Commun..

[11] Yang J., Shen X., Zhu G., Ji Z., Zhou H. (2014). ZnNi alloy nanoparticles grown on reduced graphene oxide nanosheets and their magnetic and catalytic properties. RSC Adv..

[12] Sharifi T., Gracia-Espino E., Barzegar H.R., Jia X., Nitze F., Hu G., Nordblad P., Tai C.-W., Wågberg T. (2013). Formation of nitrogen-doped graphene nanoscrolls by adsorption of magnetic γ-Fe_2_O_3_ nanoparticles. Nat. Commun..

[13] Goyal V., Balandin A.A. (2012). Thermal properties of the hybrid graphene-metal nano-micro-composites: Applications in thermal interface materials. Appl. Phys. Lett..

[14] Shahil K.M.F., Balandin A.A. (2012). Graphene–multilayer graphene nanocomposites as highly efficient thermal interface materials. Nano Lett..

[15] Nossol E., Nossol A.B.S., Guo S.X., Zhang J., Fang X.Y., Zarbin A.B.J., Bond A.M. (2014). Synthesis, characterization and morphology of reduced graphene oxide–metal–TCNQ nanocomposites. J. Mater. Chem. C.

[16] Shahil K.M.F., Balandin A.A. (2012). Thermal properties of graphene and multilayer graphene: Applications in thermal interface materials. Solid State Commun..

[17] Wang W., He D., Duan J., Wang S., Peng H., Wu H., Fu M., Wang Y., Zhang X. (2013). Simple synthesis method of reduced graphene oxide/gold nanoparticle and its application in surface-enhanced Raman scattering. Chem. Phys. Lett..

[18] Wang X., Huang P., Feng L., He M., Guo S., Shen G., Cui D. (2012). Green controllable synthesis of silver nanomaterials on graphene oxide sheets via spontaneous reduction. RSC Adv..

[19] Li Y., Lei C., Zeng Y., Ji X., Zhang S. (2012). Sensitive SERS detection of DNA and lysozyme based on polymerase assisted cross strand-displacement amplification. Chem. Commun..

[20] Lin S., Zhu W., Jin Y., Crozier K.B. (2013). Surface-enhanced Raman scattering with Ag nanoparticles optically trapped by a photonic crystal cavity. Nano Lett..

[21] Zhao N., Cheng X., Zhou Y., Yang M., Yang J., Zhong T., Zheng S. (2014). Synthesis of flexible free-standing silver nanoparticles-graphene films and their surface-enhanced Raman scattering activity. J. Nanopart. Res..

[22] Abell J.L., Driskell J.D., Zhao Y. (2014). Controllable and reversible hot spot formation on silver nanorod arrays. Chem. Commun..

[23] Tang X.Z., Cao Z. W., Zhang H.B., Liu J., Yu Z.Z. (2011). Growth of silver nanocrystals on graphene by simultaneous reduction of graphene oxide and silver ions with a rapid and efficient one-step approach. Chem. Commun..

[24] Murphy S., Huang L., Kamat P.V. (2013). Reduced graphene oxide–silver nanoparticle composite as an active SERS material. J. Phys. Chem. C.

[25] Georgakilas V., Otyepka M., Bourlinos A.B., Chandra V., Kim N., Kemp K.C., Hobza P., Zboril R., Kim K.S. (2012). Functionalization of graphene: Covalent and non-covalent approaches, derivatives and applications. Chem. Rev..

[26] Ren W., Fang Y., Wang E. (2011). A binary functional substrate for enrichment and ultrasensitive SERS spectroscopic detection of folic acid using graphene oxide/Ag nanoparticles hybrids. ACS Nano.

[27] Feng H., Cheng R., Zhao X., Duan X., Li J. (2013). A low-temperature method to produce highly reduced graphene oxide. Nat. Commun..

[28] Kumar S.V., Huang N.M., Lim H.N., Zainy M., Harrison I., Chua C.H. (2013). Preparation of highly water dispersible functional graphene/silver nanocomposite for the detection of melamine. Sens. Actuators B.

[29] Zhang Z., Xu F., Yang W., Guo M., Wang X., Zhang B., Tang J. (2011). A facile one-pot method to high-quality Ag-graphene composite nanosheets for efficient surface-enhanced Raman scattering. Chem. Commun..

[30] Barua S., Thakur S., Aidew L., Buragohain A.K., Chattopadhyay P., Karak N. (2014). One step preparation of a biocompatible, antimicrobial reduced graphene oxide–silver nanohybrid as a topical antimicrobial agent. RSC Adv..

[31] Thu T.V., Ko P.J., Phuc N.H.H., Sandhu A. (2013). Room-temperature synthesis and enhanced catalytic performance of silver-reduced graphene oxide nanohybrids. J. Nanopart. Res..

[32] Zhang Y., Yuan X., Wang Y., Chen Y. (2012). One-pot photochemical synthesis of graphene composites uniformly deposited with silver nanoparticles and their high catalytic activity towards the reduction of 2-nitroaniline. J. Mater. Chem..

[33] Liu W., Li C., Gu Y., Tang L., Zhang Z., Yang M. (2013). One-Step synthesis of β-cyclodextrin functionalized graphene/Ag nanocomposite and its application in sensitive determination of 4-nitrophenol. Electroanalysis.

[34] Dutta S., Ray C., Sarkar S., Pradhan M., Negishi Y., Pal T. (2013). Silver nanoparticle decorated reduced graphene oxide (rGO) nanosheet: A platform for SERS based low-level detection of uranyl ion. ACS Appl. Mater. Interfaces.

[35] Wang Y., Polavarapu L., Liz-Marzán L.M. (2014). Reduced graphene oxide-supported gold nanostars for improved SERS sensing and drug delivery. ACS Appl. Mater. Interfaces.

[36] Irvani S. (2011). Green synthesis of metal nanoparticles using plants. Green Chem..

[37] Alam M.N., Roy N., Mandal D., Begum N.A. (2013). Green chemistry for nanochemistry: Exploring medicinal plants for the biogenic synthesis of metal NPs with fine-tuned properties. RSC Adv..

[38] Khan M., Khan M., Adil S.F., Tahir M.N., Tremel W., Alkhathlan H.Z., Al-Warthan A., Siddiqui M.R.H. (2013). Green synthesis of silver nanoparticles mediated by *Pulicaria glutinosa* extract. Int. J. Nanomed..

[39] Khan M., Khan M., Kuniyil M., Adil S.F., Al-Warthan A., Alkhathlan H.Z., Tremel W., Tahir M.N., Siddiqui M.R.H. (2014). Biogenic synthesis of palladium nanoparticles using *Pulicaria glutinosa* extract and their catalytic activity towards the Suzuki coupling reaction. Dalton Trans..

[40] Khan M., Al-Marri A.H., Khan M., Mohri N., Adil S.F., Al-Warthan A., Siddiqui M.R.H., Alkhathlan H.Z., Berger R., Tremel W. (2014). *Pulicaria glutinosa* plant extract: A green and ecofriendly reducing agent for the preparation of highly reduced graphene oxide. RSC Adv..

[41] Malard L.M., Pimenta M.A., Dresselhaus G., Dresselhaus M.S. (2009). Raman spectroscopy in graphene. Phys. Rep..

[42] Hummers W.S., Offeman R.E. (1958). Preparation of graphitic oxide. J. Am. Chem. Soc..

